# Importance of the Choice of a Recombinant System to Produce Large Amounts of Functional Membrane Protein hERG

**DOI:** 10.3390/ijms20133181

**Published:** 2019-06-28

**Authors:** Lucie Vasseur, Thierry Cens, Renaud Wagner, Nathalie Saint, Valérie Kugler, Alain Chavanieu, Christine Ouvry, Clémence Dupré, Gilles Ferry, Jean A. Boutin

**Affiliations:** 1 Institut des Biomolécules Max Mousseron, Université de Montpellier, 34090 Montpellier, France; 2 Plateforme IMPReSs, CNRS UMR7242, Biotechnologie et Signalisation Cellulaire, Ecole Supérieure de Biotechnologie de Strasbourg, 67400 Illkirch, France; 3 PHYMEDEXP, Université de Montpellier, CNRS, INSERM, 34090 Montpellier, France; 4 PEX Biotechnologie, Chimie & Biologie, Institut de Recherches SERVIER, 78290 Croissy-sur-Seine, France; 5 Institut de Recherches Internationales SERVIER, 92150 Suresnes, France

**Keywords:** hERG, Kv11.1, *P. pastoris*, *E. coli*, HEK cells, expression, purification, functional characterization, baculovirus expression system

## Abstract

Human ether-a-gogo related gene (hERG) product is the membrane potassium channel Kv11.1, which is involved in the electrical activity of the heart. As such, it is a key player in the toxicity of many drug candidates. Therefore, having this protein at hand during earlier stages of drug discovery is important for preventing later toxicity. Furthermore, having a fair quantity of functional channels may help in the development of the necessary techniques for gaining insight in this channel structure. Thus, we performed a comparative study of methods for over-expressing a mutated but functional, hERG in different orthologous hosts, such as yeast, bacteria, insect and human cell lines. We also engineered the protein to test various constructs of a functional channel. We obtained a significant amount of a functional mutant channel from HEK cells that we thoroughly characterized. The present work paves the way for the expression of large amounts of this protein, with which protein crystallization or cryo-electronic microscopy will be attempted. This will be a way to gain information on the structure of the hERG active site and its modelization to obtain data on the pauses of various reference compounds from the pharmacopeia, as well as to gain information about the thermodynamics of the hERG/ligand relationship.

## 1. Introduction

Membrane proteins include receptors, transporters and channels. Approximately one-third of eukaryotic genes are estimated to encode membrane proteins [1]. Integral membrane proteins (IMPs) are often called “gatekeepers of the cell” [2] in reference to their crucial role in regulating the flow of information and compounds across the plasma and intracellular membranes. They are known to be involved in a number of vital cell functions, including homeostasis, growth and differentiation and neural signalling. Because of the difficulty in determining the structures of IMPs compared to globular and soluble proteins, the molecular mechanisms of action are not often described in the literature, in particular regarding the biophysics of the interaction with its ligands. Moreover, IMP research would aid in drug development, as these proteins are usual drug targets, playing key roles in a broad spectrum of human hereditary and somatic disorders and diseases [3]. Furthermore, membrane proteins are the molecular targets of roughly 40% of all FDA-approved drugs [4]. The first atomic-resolution structure of a membrane protein was published in 1985 for the photosynthetic reaction centre L-M complex [5]. In more than 40 years, the number of resolved IMP structures is still extremely low, less than 1–2% of the total protein depositions in the Protein Data Bank (PDB), which translates as 600 unique membrane protein structures in 2016 [6] and nearly 900 today (https://blanco.biomol.uci.edu/mpstruct) but it only doubled over the last 20 years [3]. The introduction and regular use of techniques linked to cryo-electron microscopy, as opposed to crystallography and X-ray diffraction may change this ratio in the coming years [7]. It is more and more common to use partially purified IMPs to obtain biophysical data [8], particularly to understand the ligand/IMP relationship at a thermodynamic level [9].

Providing large amounts of a pure protein is the first critical step toward structural and biophysical studies. Heterologous recombinant systems are commonly used to get around the presence of low amounts of IMPs in native cells. The historical production system is the bacteria *Escherichia coli*. Low cost, ease of use, availability of expression vectors and the possibility of labelling the protein are some of the well-known advantages of *E. coli* [10]. Nevertheless, a large proportion of eukaryotic membrane proteins appear to be aggregated and misfolded when expressed in bacteria [11]. This phenomenon is predicted to occur with one-third to one-half of prokaryote proteins and an even higher proportion of eukaryotic proteins [12]. The production of milligrams of pure, authentically folded protein is more often performed with yeast (i.e., *Pichia pastoris*), insect or mammalian cells rather than *E. coli*. A number of specific production strategies have been developed for each of those host systems. Interestingly, the approach to G-protein coupled receptor pharmacology helped develop a series of techniques to address as many of the problems of IMP expression and purification as possible [13,14,15]. Considerable progress has been published on the reconstitution of such proteins and their capacity, once reconstituted, to be functional [13,14,15] but >99% sample purity is still challenging in crystallization success. Such a purity standard is often far from our immediate grasp for membrane proteins. Furthermore, low expression levels are still frequently reported for IMPs [11] and it is obvious that reaching high levels of recombinant protein expression will permit to purify to a decent yield this difficult category of proteins.

The human-ether-a-go-go related gene (hERG) encodes the voltage-activated potassium channel Kv11.1 [16]. This ionic channel is predominantly involved in cardiac rhythm with crucial action in cardiomyocyte repolarization [16]. It is also expressed, though to a lesser extent, in the nervous system, jejunum and endocrine system. This channel is a non-specific target of a variety of structurally diverse drugs [17]. This unwanted blockage of hERG function by drugs can lead to long QT syndrome (LQTS), causing severe heart conditions [18,19]. In this context, regulators and pharmaceutical companies are concerned, establishing tests to detect drug-induced pro-arrhythmic risk at early phases of drug discovery. Verification that a drug candidate at its earliest stage of qualification does not bind to hERG is key to pursuing drug development. During the last decade, a considerable number of studies have focused on this channel from pharmacological and technological points of view [17,20]. The potassium permeable pore is a homo-tetrameric assembly of hERG subunits. Each 1159-amino-acid monomer constitutes a transmembrane domain containing six alpha-helices [16]. The helices from S1 to S4 are responsible for the voltage sensor activity of the channel [21,22]. The pore domain is comprised of S5 and S6 helices and a pore helix (P) between S5 and S6 [23]. The N- and C-termini contain 400 and 500 amino acids, respectively and are located in the cytoplasmic compartment [16,24]. Similar to other IMPs, hERG structural studies have been hindered by several difficulties, such as recombinant production in large amounts as well as solubilizing and purifying a functional stable protein. Interestingly, the most recent work on the hERG structure used cryo-electron microscopy after production in a HEK cell line infected by a virus encoding a truncated version of the protein [24]. In 2013, Hausammann and Grütter [25] published a study in which they engineered a minimal construct based on the hERG sequence. This construct contained the whole membrane domain of hERG with six alpha-helices but omitted both the N and C terminus cytoplasmic extremities. After truncation, an artificial tetramerization domain in the C terminus, the GCN4-LI leucine zipper [26], was added. This 33-amino-acid peptide forms an extremely thermostable tetrameric parallel coiled-coil. This minimal chimeric channel was shown to be functional by recording currents using two electrode voltage clamps in xenopus oocytes [25].

In the present study, we present several strategies for the expression, purification, reconstitution and characterization of the chimeric version of hERG(S1-coil) described by Hausammann and Grütter [25]. We started by generating and producing the protein in three recombinant systems. The amount of total functional channel produced was investigated. We also tested the capacity of non-ionic and zwitterionic detergents to extract the channel from membranes before proceeding to its purification. Purified hERG channels reconstituted in planar lipid bilayers were functionally evaluated by electrophysiology.

## 2. Results and Discussion

For biophysical in vitro studies and structural investigations of proteins, a large amount of material in the range of milligrams is usually required. With membrane proteins, recombinant systems over-expressing the gene of interest are required because of their typical low abundance in their natural hosts [27]. Because no universal system is readily available for this purpose, our first task was to identify the best recombinant sequence to introduce into the most appropriate production system. Our attempts to produce the human hERG(S1-coil) channel in bacteria failed at the initial step of this study (see details below, Section 2.2), so we selected a panel of three representative expression systems shown to be efficient for over-expressing several membrane proteins: the eukaryotic microorganism *P. pastoris* [28,29,30,31], the Sf9 insect cell line [32] and the human HEK cell line used for transient expression or stable and inducible expression [33,34]. We also fused various tag sequences to the channel cDNA based on their proven benefits with regard to detection, purification and/or protein stability. For a better understanding of our strategy, we report in Figure S1 the sequence of the wild type hERG and in Figure S2, the actual sequences we used in the present work.

### 2.1. Analysis of hERG(S1-coil) Function in Xenopus Oocytes

**Figure 1:**
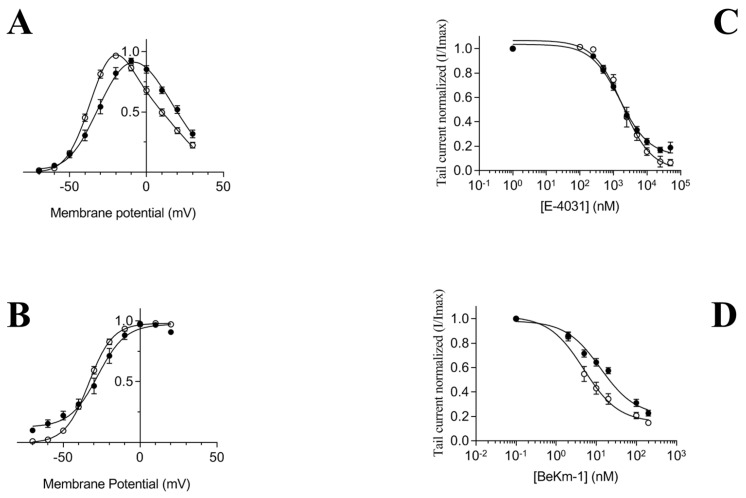
Voltage activation and pharmacological properties of hERG(S1-coil) recorded in Xenopus oocytes. (**A**). Current-voltage relationship for hERG-wt and hERG(S1-coil). Currents measured at the end of a 4 seconds long depolarizing pulse (from −70 to 30 mV) were normalized to the maximal current for hERG-wt (*n* = 21) and hERG(S1-coil) (*n* = 19). (**B**). Steady-state voltage-dependence of activation. The tail currents at −50 mV were normalized to the peak tail current, plotted against the amplitude of the depolarizing pulse and fitted with a Boltzmann function to estimate the potential for half-activation (V1/2) and the slope value (k) for hERG-wt (V1/2 = −33.3 ± 0.9 mV, k = 7.5 ± 0.4 mV, *n* = 21) and hERG(S1-coil) (V1/2 = −27.7 ± 2.3 mV, k = 6.0 ± 0.4 mV, *n* = 19). Note that the values obtained with hERG(S1-coil) are significantly different from those obtained with hERG-wt (*p* < 0.01). (**C**). Inhibition curves for hERG-wt (*n* = 9) and for hERG(S1-coil) (*n* = 13) by E-4031 determined by measuring the tail current at −40 mV in the presence of increasing concentration of E-4031 and normalized to the control current measured in the absence of drug at a voltage of −40 mV after 2 seconds of depolarization at +20 mV. Curves are fitted with a logistic function. (**D**). Inhibition curves for hERG-wt (*n* = 7) and for hERG(S1-coil) (*n* = 8) by the BeKm-1. hERG wild type = empty circles; hERG(S1-coil) = filled squares.

First, we confirmed activation gating of the chimera hERG(S1-coil) channel using the voltage-pulse protocol in *Xenopus laevis* [25]. As shown previously, hERG(S1-coil) has very similar gating characteristics as wild type hERG. The Boltzmann fit of the I-V data revealed a half-maximal activation (V_1/2_) of −27.7 ± 2.3 mV for hERG(S1-coil) and −33.3 ± 0.9 mV for hERG-wt and a slope (*k*) of 6.0 ± 0.4 mV for hERG(S1-coil) and 7.5 ± 0.4 mV for hERG-wt (Figure 1A,B). In addition, inhibition by E-4031 and the BeKm-1 toxin had close IC_50_ for hERG(S1-coil) (24.87 ± 3.77 nM with BeKm-1 and 2.3 ± 0.2 µM with E-4031) and hERG-wt (21.67 ± 2.76 nM with BeKm-1 and 2.5 ± 0.3 µM with E-4031) (Figure 1C,D). These results are comparable to those from the literature [25,35] and demonstrate that the replacement of hERG inherent tetramerization domains by a leucine zipper motif maintains oligomeric stability and channel function.

### 2.2. Comparison of Functional hERG(S1-coil) Expression in Different Systems

Four expression protocols were followed to produce the hERG(S1-coil) chimera channel. The Sf9 (*Spodoptera frugiperda*) insect cell line, *P. pastoris and* mammalian HEK cell line (transient or stable expression) were tested according to the protein constructs described in Table 1. For expression in Sf9 cells, the sequence 2Strep-3C-hERG(S1-coil)-TEV-6His was integrated into baculovirus, followed by viral infection. In the yeast *P. pastoris*, the hERG(S1-coil)-10His production was induced after integrative transformation. Finally, two different approaches were tested for expression in the HEK cells: FreeStyle transient transfection and the generation of an inducible stable cell line by lentiviral transduction. For expression in HEK cells, the hERG(S1-coil)-TEV-SBP-6His construct bearing a N598Q mutation at the glycosylation site was produced to increase the homogeneity of over-expressed protein.

Moreover, we experimented several expression conditions in various *E. coli* strains. The DNA sequence of hERG(S1-coil) has been codon-optimized for expression in *E. coli* and integrated into the pET32a vector (Novagen) with a TEV sequence and a 6His tag in C-terminal. The recombinant plasmid has been transformed into different three *E. coli* strains: BL21(DE3)pLysS (Invitrogen), Rosetta-gami(DE3)pLysS (Novagen) and C41(DE3)pLysS (Lucigen). Expression was induced at an OD600 between 0.4 and 0.6 in LB medium by the addition of 0.1 or 1 mM of IPTG (isopropyl β-d-1-thiogalactopyranoside). We carried out several time courses at different temperatures, such as 15, 28 and 37°C. Nevertheless, SDS-PAGE and Western Blot analysis of both the total and the insoluble fractions of bacteria did not indicated any presence of the protein of interest (not shown).

**Figure 2:**
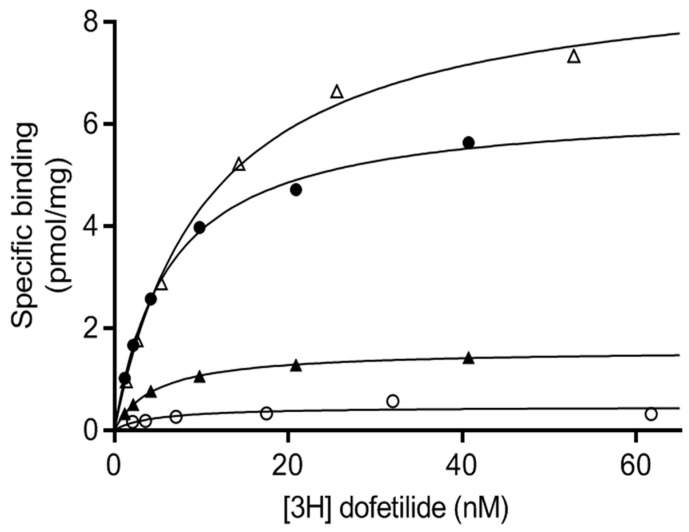
Characterization of the binding properties of [^3^H]-dofetilide to hERG(S1-coil) expressed in three recombinant systems. The chimeric hERG(S1-coil) channel was expressed in the yeast *P. pastoris*, Sf9 insect and mammalian HEK293 cells (transient transfection and stable and inducible cell lines). Membranes were prepared for each system. Saturation ligand binding experiments with [^3^H] dofetilide were performed using 10 µg of total membrane protein. Specific binding was calculated from total and non-specific measurements in triplicate (unspecific values are comprised between 0.5 and 1 pmol/mg). All error bars are all less than 0.6 pmol/mg. Empty triangles = *P. pastoris*; Filled circles = HEK stable; Filled triangles = Sf9; Empty circles = HEK transient.

After expression in each system, the level of functional channel was quantified by ligand-binding studies on membrane preparations using [^3^H]-dofetilide as the tracer ligand (Figure 2). The saturation format used in these binding studies allowed the determination of a B_max_ value for each sample, which represents the maximal amount of ligand required to saturate a population of receptors, and thus an estimation of the number of receptors present in the sample. The K_D_ parameter, which corresponds to the ligand concentration needed to saturate half of the binding sites, reflects on the affinity of the receptor for its ligand (Table 2). While the K_D_ values are globally consistent between the different expression systems tested, the calculated B_max_ is particularly low in the case of production in Sf9 cells and transient HEK (0.70 ± 0.10 and 0.73 ± 0.04 pmol/mg of membrane proteins, respectively). The generation of a stable cell line with the capacity to express hERG(S1-coil) by doxycycline induction resulted in a 7-fold enhancement of functional channels in membranes, with 5.04 ± 0.2 pmol/mg of membrane proteins calculated for the B_max_ value. Integration of the protein sequence into the cell genome allows stable genetic transmission in each cell, as the plasmid tends to be lost after transitory transfection. Second, the stable cell line has the capacity to be induced in a way that avoids the risk of cell toxicity usually reported in constitutive expression [36]. Surprisingly, the best [^3^H]-dofetilide binding B_max_ value of 12.0 ± 0.3 pmol/mg of membrane proteins was recorded after production in *P. pastoris* (see Table 2 for details). Although the HEK cell line is the most homologously similar system to the physiological environment of the hERG channel, *P. pastoris* allowed the higher expression of functional channel. We focused on both *P. pastoris* and the stable HEK cell line in subsequent steps.

The relative variability of the B_max_ and K_D_ values that is observed between the different batches in Table 2 is a common issue with the overexpression of membrane proteins. Even if such an observation is poorly documented in the literature, some studies have already illustrated this aspect (see Logez et al. [13]). Such heterogeneity is likely to be related to the overloading of the cell machineries during the massive production of membrane proteins that is potentially triggering unfolding response pathways. These events may then vary from batch to batch, ending with variable levels of functional proteins. This is particularly the case with mammalian cells expression systems where some factors that are less controlled (culturing medium lots, number of cell passes) may have a significant impact on these aspects.

### 2.3. Total Expression of hERG(S1-coil) in Different Cell Membranes

**Figure 3:**
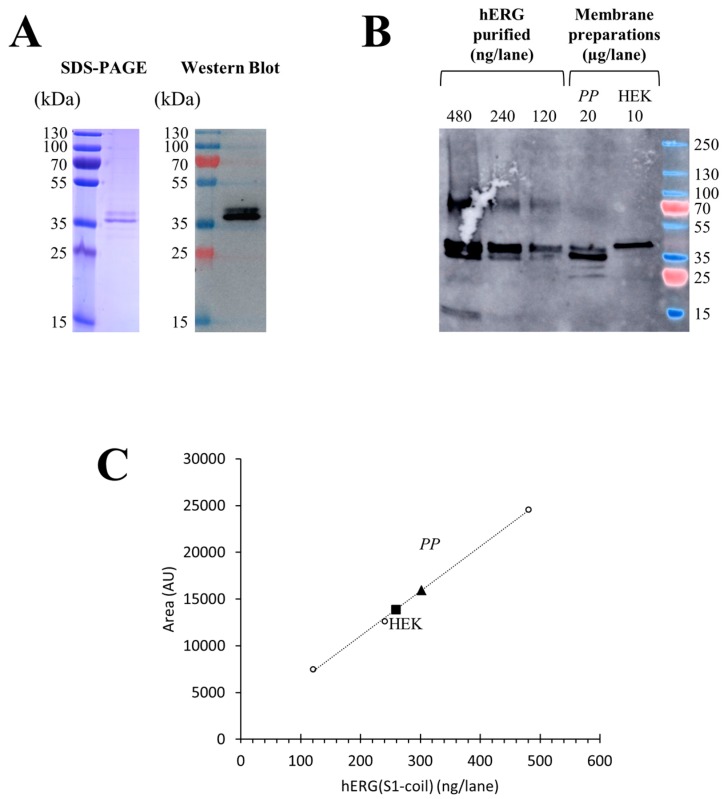
Quantification of hERG in *P. pastoris* and HEK membranes. **A.** hERG(S1-coil) expressed in *P. pastoris* was solubilized in 1% dodecylphosphocholine (DPC) and purified on a TALON^®^ resin. The presence and purity of the protein was validated by SDS-PAGE (lane 1) and Western blot (lane 2). The purified protein was quantified by the BCA test. **B**. Increasing quantities of purified proteins and membrane preparations of *P. pastoris* and HEK were studied by Western blot using an anti-hERG antibody. **C**. hERG can be quantified in membranes of *P. pastoris* (triangle) and HEK (square) cells after fitting the linear equation between the amount of purified hERG(S1-coil) from A used as a standard and the fluorescent area on Western blot (R² = 0.9986).

The total production yields of hERG(S1-coil) were investigated by quantitative Western blot on membranes prepared from *P. pastoris* and stable HEK cells. For quantification after protocol optimization, 20 µg of total membrane protein prepared from *P. pastoris* and 10 µg of total membrane protein prepared from HEK cells were analysed by comparison with increasing quantities from 120 to 480 ng of purified hERG(S1-coil) channel (Figure 3B). Thus, the signal intensity on Western blot is directly linked to the quantity of channel without discrimination of functional or non-functional, as we worked under denaturing conditions. Purified sample was obtained from *P. pastoris* membranes expressing hERG(S1-coil), solubilized in dodecylphosphocholine (DPC) and purified on TALON^®^ resin. The purity of the purified protein was checked by SDS-PAGE (Figure 3A) and the protein concentration of the sample measured by BCA assay.

Notably, expression of the non-mutated channel at the N-glycosylation site in *P. pastoris* resulted in a double band. In contrast, the channel mutated at N598 and produced in HEK cells resulted in a single band (Figure 3B). This typical feature of hERG has already been described in previous studies [37,38]. Regarding this experiment, the calculated amount of hERG(S1-coil) channel in *P. pastoris* was 15.1 ± 3.7 µg/mg of total membrane proteins (*n* = 3) and 25.9 ± 4.3 µg/mg of total proteins in HEK membranes (*n* = 3). In conclusion, stable HEK cells produce higher amounts of hERG(S1-coil) protein than *P. pastoris* based on the analysis of total expression and without discrimination of functionality.

### 2.4. Deduction of the Percent of Activity in Membranes

Finally, we wanted to quantify folded and misfolded protein in membranes by comparing the total quantity of proteins in Western blot with the amounts of functional channel in binding assays. The percent of activity was defined as the ratio between functional and total channel in membranes. Approximately 10% of the channels in *P. pastoris* membranes and 5% in stable HEK cells membranes were in a functional state (Table 3). The percent of functional channels in membranes from stable HEK cells was lower due to a higher level of total channel production compared to membranes from *P.* pastoris. Conversely, with a smaller total amount of channels and a higher amount of functional channels, *P. pastoris* afforded the best balance between the expression of receptors and the number of functional receptors. Thus, considering our goal of producing large amounts of active hERG(S1-coil) and evaluating the functional part of the protein in the total production, *P. pastoris* seems to be the best recombinant system.

### 2.5. Solubilization of hERG(S1-coil) in Non-Ionic and Zwitterionic Detergents

After production of a membrane protein, another challenging step is membrane extraction to individualize the protein before purification. The critical point is to get the protein out of its lipid environment while maintaining a native conformational state [39]. Thus, detergents have been widely used for decades to solubilize membrane proteins; they have the ability to substitute lipids around the hydrophobic domains of the protein via micelle arrangement [40]. Detergents are classified into three main classes: non-ionic, zwitterionic and ionic. Ionic detergents, including sodium dodecyl-sulphate (SDS), have a denaturing effect on membrane proteins by disrupting both inter- and intra-molecular protein-protein interactions [41]. For this reason, we did not use this class of molecules. Zwitterionic detergents are softer because of their neutral global charge. For example, Fos-choline-14 was recently shown to be the most suitable detergent for solubilizing the Kir6.2 potassium channel by maintaining the native tetrameric structure [42]. Nevertheless, these molecules, including DPC, can often disrupt protein-protein interactions. The non-ionic detergents, including dodecylmaltoside (DDM), are a non-denaturing group of molecules that only disrupt protein-lipid and lipid-lipid interactions. In the literature, most of the membrane proteins are extracted and purified in non-ionic detergents, such as DDM [43].

**Figure 4:**
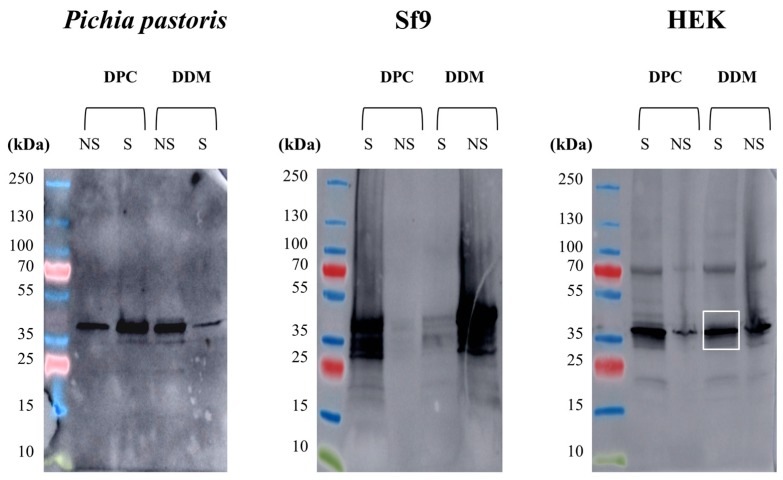
Detergent extraction of hERG from the membranes of three recombinant systems. Membranes were prepared from yeast *P. pastoris*, Sf9 insect cells or stable mammalian HEK cells expressing hERG(S1-coil). A sample of each membrane containing 20 µg of total protein for *P. pastoris* and HEK cells or 1000 µg of protein for Sf9 cells was incubated with 1% dodecylmaltoside (DDM) or 1% DPC. Solubilized (S) and non-solubilized (NS) fractions were studied by Western blot with an anti-His antibody.

After production in three recombinant systems, the potency of two detergents for extracting hERG(S1-coil) from membranes of *P. pastoris*, Sf9 and stable HEK cells was evaluated. Both the non-ionic detergent DDM and zwitterionic detergent DPC were tested. Results are given in Figure 4. Interestingly, we found that hERG(S1-coil) expressed in three recombinant systems had different solubilization properties. Though hERG(S1-coil) produced in HEK cells can be solubilized in both zwitterionic and non-ionic detergents, the channel produced in *P. pastoris* or Sf9 cells is only solubilized in the harsh detergent DPC but not in the soft detergent DDM. This differential behaviour is likely to be related to lipid variations in the membrane composition between the three cell hosts. Regarding the solubilization with DDM, that more likely preserves the tetrameric organization of the channel, stable HEK cells could be a better strategy for production, from which the protein could be functionally extracted by DDM, despite the percent of functional channels being lower than *P. pastoris*.

### 2.6. Channel Function after Extraction with Detergent

**Figure 5:**
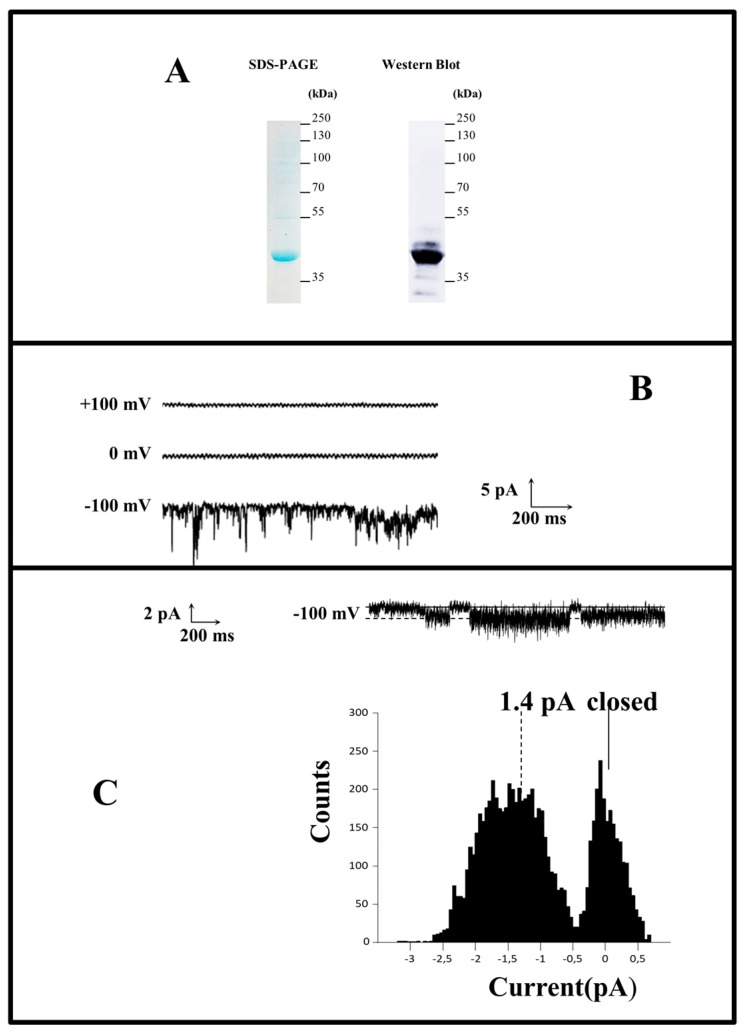
hERG channel function in lipid bilayers after HEK expression and DDM solubilization. **A.** The purified hERG(S1-coil) was overexpressed in stable HEK cell line, solubilized in 1% DDM and the resulting supernatant was purified on a Strep affinity resin and eluted with 2 mM biotin. **B**. hERG(S1-coil) multichannel currents recorded at an applied potential of +100, 0 and −100 mV. **B.** Single hERG(S1-coil) channel currents recorded at an applied potential of −100 mV. **C**. Current trace and related point-amplitude histogram.

Thus, to investigate the channel function after membrane extraction in detergent, we purified the protein from *P. pastoris*, Sf9 or stable HEK membranes and integrated the purified channel into reconstituted bilayer membranes to record currents. We wanted to determine whether previous solubilization in non-ionic (DDM) or zwitterionic (DPC) detergents interferes with the native conformation and function of the channel. The differences between DDM and DPC in terms of their extraction potency and the resulting solubilized protein functionality is a common observation that has already been reported (see Chipot et al. [44] for a review). As illustrated in Figure 5A, in the case of HEK membranes solubilized and purified in DDM, the hERG(S1-coil) channel was able to open after depolarization at a negative potential of −100 mV. In addition, both single and multiple-channel activities were only observed at negative potentials.

As reported in the literature, no outward currents were observed at positive voltages [45]. Moreover, samples pre-incubated with 10 µM of BeKm-1 toxin did not have any current at −100 mV (data not shown). This confirms the identity of the ionic channel recorded in this experiment. By focusing on hERG(S1-coil), single-channel stepwise currents were recorded with an average of 1.4 pA (Figure 5B). These data are in agreement with hERG-wt current records in the literature [46]. In contrast, when hERG(S1-coil) channels from *P. pastoris* and Sf9 membranes solubilized in DPC were integrated into bilayer membranes, no electrophysiological activity was detected (data not shown). These results demonstrate that the channel is in an activated-open state after extraction in DDM but seems to be mostly non-functional after extraction in DPC. This result is in agreement with our hypothesis and confirms that it is preferable to produce hERG(S1-coil) in stable HEK cells to allow membrane solubilization in DDM and then purify a functional channel for structural and/or biophysical characterization.

## 3. Conclusions

Access to fair amounts of integral membrane proteins is imperative in modern pharmacology research. Thus, different accessible systems susceptible to producing a functional protein were tested. Because of the absence of expression in E. coli, this system has been early abandoned. Then, expression in Sf9 showed really poor expression of functional protein. Interestingly, we found that the mutated/truncated hERG protein was expressed in the largest amount in the *P. pastoris* system in a functional state. But conversely, no extraction with the smooth detergent DDM was possible with this recombinant system and extraction with the harsh detergent DPC resulted in a non-functional channel. In contrast, lower amounts of hERG channel were produced with the stable and inducible HEK cell line but the possibility of extraction with DDM offered a functional purified channel. In addition, we demonstrated that the generation of a cell line is a way to significantly increase the amount of expression of the protein of interest compared to transient expression in HEK cells. We were able to purify approximately 100µg of the channel from 1 L of culture from our cell line (2.10^8^ cells). Thus, we grossly estimate that approximately 15 L of HEK culture (roughly 5.10^9^ cells) should be necessary to prepare a batch of 1 mg of purified hERG(S1-coil). This study illustrates difficulties commonly met for heterologous expression of a membrane protein. The balance between growing quantities and maintaining the activity (avoiding misfolding and aggregation) needs to be finely controlled and dictates the selection of one host over the other. In this context, this new approach paves the way for gaining information on structure and the biophysical interactions between any ligands and the purified channel. Although recent studies provide new elements on hERG function and structure, several questions are still unanswered to understand the mechanisms of drug binding.

In summary, hERG was highly expressed in *P. pastoris* but solubilization led to a dead channel; there was no expression in *E. coli* while expression in mammalian cells (HEK cells), even if at relatively low level, led to a functional purified channel.

## 4. Materials and Methods

### 4.1. Materials

Astemizole was purchased from Sigma Aldrich (Saint-Quentin Fallavier, France), E-4031 from Cayman Chemical (Ann Arbor, MI, USA), recombinant toxin BeKm-1 and anti-hERG(extracellular) antibody from Alomone (Jerusalem, Israel) and [^3^H]-dofetilide from Perkin Elmer (Roissy-en-France, France). Detergents n-dodecyl-β-d-maltopyranoside (DDM) and n-dodecylphosphocholine (DPC) were ordered from Anatrace (Maumee, OH, USA). FreeStyle™ 293-F cells were purchased from ThermoFisher Scientific (Villebon, France). A stable FreeStyle™ 293-F cell line expressing hERG(S1-coil) after doxycycline induction was also ordered from Vectalys (Toulouse, France).

### 4.2. Molecular Biology

The hERG full-length sequence in pcDNA3.1 plasmid was provided by Dr. Thierry Cens from IBMM (Montpellier, France). A second plasmid, pEX-A2, containing the coiled-coil sequence was ordered from Eurofins Genomics. As described by Hausammann and Grütter [25], the truncated hERG sequence from amino acids 362 to 675 was cloned by PCR using the following primers: forward F1, CATGAAGATAAAGGAGCGAACCCAC; and reverse R1, CATCCTGGAACCAGATCC- GCCTGTGTGGTATCGGGCTGTGC. The coiled-coil sequence was cloned using the following primers: forward F2, GCACAGCCCGCTACCACACAGGCGGATCTGGTTCCAGGATG; and reverse R2, CTATCTTTCCCCTAACAACTTCTTTATGC. Both were linked by overlapping PCR using oligonucleotides F1 and R2. The chimeric construct was checked by sequencing. For expression in *Xenopuslaevis* oocytes, the hERG(S1-coil) sequence was subcloned into a pCMV-PL10 vector, with the Alfalfa Mosaic Virus (AMV) sequence immediately before the start codon and the 3′-UTR sequence of the *X. laevis* -globin gene immediately after the stop codon. Capped cRNA transcripts coding for hERG(S1-coil) were obtained from linearized plasmid using the mMessagemMachine T7 transcription kit (Thermo Fisher), following the manufacturer’s instructions. The concentrations were adjusted to 1 µg/µL. For high level production in different recombinant systems, optimized sequences were ordered from GeneCust (Ellange, Luxembourg). Selected plasmids and additional tags are referenced in Table 1.

### 4.3. Electrophysiology in Xenopus Oocytes

Stage V–VI oocytes were removed from female Xenopus under tricaine anaesthesia. Oocytes were defolliculated by treatment with 1 mg/mL collagenase type 1A (Sigma Aldrich) under agitation at room temperature in OR2 solution (82.5 mM NaCl, 2 mM KCl, 1 mM MgCl_2_, 5 mM HEPES, pH 7.2). After intensive washing with OR2 solution, oocytes were injected with RNA preparation (about 30 nL per oocyte) and recorded 2–5 days after injection. Currents were recorded at room temperature using the two-electrode voltage clamp method. Electrodes were pulled from borosilicate glass and filled with 3 M KCl. Currents were measured by a Geneclamp 500 amplifier (Molecular Devices, San Jose, CA, USA) and digitized by a Digidata 1200 converter (Molecular Devices) using Clampex 5 software (Molecular Devices). Oocytes were clamped at −80 mV and leak currents subtracted online using a P/3 protocol of hyperpolarizing pre-pulses. The external solution, ND-hERG, has the following composition: 96 mM NaCl, 3 mM KCl, 0.5 mM CaCl_2_, 1 mM MgCl_2_, 5 mM HEPES, pH 7.4. Inhibitors were diluted in ND-hERG solution and superfused through the recording chamber at a rate of 1 mL/min with increasing concentrations: from 100 pM to 200 nM for BeKm-1 and from 1 nM to 50 µM for E-4031. Currents were elicited by a two-step depolarization protocol from a holding potential of −80 mV with voltages ranging from −70 to −30 mV in 10 mV increment during 4 s and then back to −50 mV for 2 s. We determined the concentration to obtain the half maximal inhibition (IC_50_) by measuring the peak tail current recorded during the second pulse at −50 mV in the presence of increasing concentrations of inhibitor and normalized the value to the current measured in the absence of inhibitor. Curves were fitted with a logistic function (Prism, GraphPad). Data are represented as mean ± SEM of n oocytes. The significance of the difference between two results was determined using the unpaired Student’s test set at the 0.01 level.

### 4.4. Production in P. pastoris

The hERG(S1-coil) sequence was subcloned into a pPIC9K vector with a 10-histidine tag at the 3′ end of the construct. *P. pastoris* SMD1163 cells were transformed with the plasmid. Selection for His+ transformants was carried out by Yeastern Blot [29]. One clone was selected for further study. A single colony was grown overnight in BMGY (1% (*w*/*v*) yeast extract, 2% (*w*/*v*) peptone, 1.34% (*w*/*v*) yeast nitrogen base without amino acids, 1% (*w*/*v*) glycerol and 0.1 M phosphate buffer, pH 6.0 at 30 °C with shaking at 250 rpm until an optical density at 600 nm (OD_600_) between 4 and 5 (arbitrary units). The hERG(S1-coil) expression was induced at +22 °C for 20 h in BMMY medium 1% (*w*/*v*) yeast extract, 2% (*w*/*v*) peptone, 1.34% (*w*/*v*) yeast nitrogen base without amino acids, 0.5% (*v*/*v*) methanol and 0.1 M phosphate buffer, pH 6.0) containing 2.5% dimethylsulfoxide (DMSO). Cells were harvested by centrifugation and stored at −80 °C until purification.

### 4.5. Production in Sf9 Insect Cells

The hERG(S1-coil) sequence optimized for expression in insect cells was subcloned into pFB1. A double-streptavidin tag and 3C cleavage site were added at the 5′ end of the construct and a TEV (Tobacco Etch Virus) cleavage site and 6-histidine tag added at the 3′extremity (Table 1). Following the Bac-to-Bac^TM^ method (Invitrogen, Life Technologies, ThermoFischer Scientific), the recombinant vector was transferred to competent *E. coli* DH10Bac cells to produce recombinant bacmid by homologous recombination. Viral stock was obtained by transfection of Sf9 cells with the recombinant bacmid. Finally, a culture of Sf9 insect cells at a density of 4 × 10^6^ cells/mL in suspension in EX-CELL^®^420 Serum-Free medium (SigmaAldrich) was infected by the viral stock encoding hERG(S1-coil) at a concentration of 1:500 (*v*/*v*) for 48 h at 28 °C with shaking. Cells were harvested by centrifugation and stored at −80 °C until purification.

### 4.6. Production in HEK Cells

For transient production, the synthetic gene corresponding to hERG(S1-coil) was integrated into a pCI-neo plasmid containing a TEV cleavage site, streptavidin binding peptide (SBP) tag and 6-histidine tag at the 3′ end of the chimeric construct. A mutation was inserted by replacing asparagine (N) at position 598 of the hERG amino acid sequence with a glutamine (Q). FreeStyle™ 293-F cells were transfected by electroporation. The stable cell line was generated by lentiviral transduction of HEK293FS EF1-tTS+EF1-TetOn3G-IRES-Neo with a lentivirus, rLV.TRE3G, containing hERG(S1-coil) with a TEV cleavage site, SBP tag and 6-histidine tag at the 3′ end of the chimera construct. An N598Q mutation was also inserted. After clonal selection, cells were cultivated in Freestyle 293 media (Gibco, ThermoFisher Scientific) complemented with 1% H-T supplement (Gibco) and 0.25% penicillin-streptomycin. For production, cells were seeded at 200,000 cells/mL and incubated at +37 °C in 8% CO_2_ at 125 rpm. Production of the protein of interest was induced with 10 ng/mL doxycycline. After 24 h of incubation, cells were pelleted by centrifugation.

### 4.7. Preparation of Crude Membranes

For membrane preparation of yeast, pellets were resuspended in a lysis buffer containing 50 mM Tris, pH 7.4, 500 mM NaCl, 10% glycerol, 1 mM ethylenediaminetetraacetic (EDTA) and 1 mM phenylmethylsulfonyl fluoride (PMSF). Glass beads (0.5 mm; SigmaAldrich) were added to the mixture. Cell lysis was carried out using FastPrep 24 (MP Biomedicals, Illkirch, France) with alternating stirring and cooling cycles followed by 5 min of centrifugation at 5000 *g* and +4 °C. The supernatant was then ultracentrifuged at 100,000 *g* for 30 min at +4 °C. The pellet was resuspended in membrane buffer (50 mM Tris, pH 7.4, 500 mM NaCl, 10% glycerol). For insect and mammalian cells, the following protocol was carried out to prepare membranes. Cellular pellets were suspended in lysis buffer containing 10 mM HEPES (pH7.4), 1 mM EDTA and 1 mM PMSF using a Dounce homogenizer. The mixture was centrifuged for 10 min at 1000 *g* and +4 °C. The supernatant was then ultracentrifuged at 100,000 *g* for 30 min at +4 °C. The pellet was resuspended in membrane buffer (50 mM HEPES, pH 7.4, 120 mM NaCl, 10% glycerol) with a ratio of 1 mL of membrane buffer for 1.5 × 10^8^ cells. After crude membrane preparation and for each production system, a BCA protein assay (Thermo Fisher) was performed following the manufacturer’s instructions.

### 4.8. Radiolabeled Ligand Binding Assay

Crude membranes were used to assess the capability of recombinant hERG(S1-coil) to bind one of its ligands, dofetilide. Aliquots of 10 µg of total membrane proteins in a total volume of 100 µL of incubation buffer (10 mM HEPES, pH 7.5, 130 mM NaCl, 60 mM KCl, 0.8 mM MgCl_2_, 1 mM NaEGTA, 10 mM glucose, 0.1% BSA) were mixed with [^3^H]-dofetilide at concentrations ranging from 1 nM to 40 nM. Non-specific binding was determined in the presence of 10 µM of astemizole [47]. After 2 h of incubation at room temperature, the mixtures were filtered on 96-well Unifilter GF/B plates pre-soaked in poly-ethyleneimine (PEI) and washed six times with 1 mL ice cold wash buffer (10 mM Tris-HCl, pH 7.4, 130 mM NaCl, 5 mM KCl, 0.8 mM CaCl_2_, 0.1% BSA). Filter-bound ligand was counted using a TopCount (Perkin Elmer) scintillation counter. Total binding and non-specific binding were determined with these measures and specific binding deduced. A resulting graph was drawn with Prism (v8, GraphPad, San Diego, CA, USA) using the Sigmaplot non-linear regression tool, ligand binding and one site saturation (f = B_max_ x abs(x)/(K_d_ + abs(x))) to estimate binding affinity (K_d_) and capacity (B_max_).

### 4.9. Protein Quantification by Western Blot

To quantify total protein produced in *P. pastoris* and HEK membranes, a sample corresponding to 10 or 20 µg of total protein were analysed by polyacrylamide gel electrophoresis and transferred onto a nitrocellulose membrane. hERG(S1-coil) was specifically labelled with an anti-hERG extracellular primary antibody at a dilution of 1:500 (*v*:*v*) followed by a secondary fluorescent anti-rabbit-HRP antibody (Sigma Aldrich).

A range of concentrations with purified hERG(S1-coil) at known concentration determined by the BCA assay were also deposited on gel. The hERG(S1-coil) concentration in membrane samples was calculated using ImageJ (https://imagej.nih.gov/ij/) after determination of the relationship between hERG(S1-coil) concentration and band intensity on Western blot.

### 4.10. Protein Solubilization and Purification

Crude membranes prepared from *P. pastoris*, Sf9 or stable HEK cells expressing hERG(S1-coil) were solubilized in 50 mM HEPES, pH 7.5, 300 mM KCl containing 1% DPC or DDM and 0.2% cholesteryl-hemisuccinate (CHS, Sigma Aldrich) at slow rotation at +4 °C for 1 h. Samples were centrifuged at 25,000 *g* for 15 min at +4 °C. For the purification of hERG(S1-coil) overexpressed in *P. pastoris* in DPC, the supernatant was incubated with Co^++^ affinity resin (TALON^®^ Metal Affinity Resin, Clontech, Saint-Germain-en-Laye, France) at a ratio of 1 mL resin for 30 mL of supernatant with 20 mM imidazole at slow rotation at +4 °C for 1 h. After transferring to a column and washing the resin with 15 CV of 50 mM HEPES, pH 7.5, 300 mM KCl, 50 mM imidazole, 0.1% (*w*/*v*) DPC and 0.02% (*w*/*v*) CHS, hERG(S1-coil) was eluted with 250 mM imidazole in the same buffer. For purification of hERG(S1-coil) overexpressed in Sf9 cells in DPC, the supernatant was diluted 2-fold in 50 mM HEPES, pH 7.5 and transferred to a Strep-Tactin column (Strep-TactinSuperflow, IBA, Göttingen, Germany) at a ratio of 1 mL resin for 100 mL supernatant. The resin was washed with 15 CV of 50 mM HEPES, pH 7.5, 300 mM KCl, 0.1% (*w*/*v*) DPC and 0.02% (*w*/*v*) CHS and elution was performed by the addition of 2.5 mM D-desthiobiotin (IBA) in the same buffer. Purification of hERG(S1-coil) overexpressed in stable HEK cells was carried out in DDM. The supernatant was diluted 2-fold in 50 mM HEPES, pH 7.5 and transferred to a column with streptavidin resin (High Capacity Streptavidin Agarose Resin, Life Technologies, Thermo Fischer Scientific) at a ratio of 1 mL resin for 100 mL supernatant. The resin was washed with 15 CV of 50 mM HEPES, pH 7.5, 300 mM KCl, 0.1% (*w*/*v*) DDM and 0.02% (*w*/*v*) CHS and elution was performed by the addition of 2 mM biotin (Sigma) in the same buffer. Purified samples were analysed by SDS-PAGE and Western blot and protein concentrations determined by BCA assay.

### 4.11. Electrophysiology on Lipid Bilayers

Planar lipid bilayers were formed at room temperature by painting a solution of azolectin (Type IVS, Sigma Aldrich) at 45 mg/mL in decane across a 200 µm aperture in a polysulfonate cup (Warner Instruments, Hamden, CT, USA) separating two chambers. The *trans* chamber compartment was connected to the headstage input of a bilayer voltage clamp amplifier (BC-525D, Warner Instruments). The *cis* chamber was held at virtual ground. Voltages were applied to the planar lipid bilayers through Ag-AgCl electrodes connected to the chambers via agar/KCl bridges. The solution in both chambers was 120 mM KCl in 10 mM HEPES, pH 7.3. A total of 500 ng of chimeric hERG protein was added in the *cis* chamber and the resulting channel activity recorded for at least 2 min. For analysis, the data were filtered to 1 kHz, digitized at 4 kHz and collected on a Pentium computer using Axoxcope10 and Digidata 1440A (Molecular Devices, San José, CA, USA).

## Figures and Tables

**Table 1:** Overview of hERG(S1-coil) channel production in different recombinant systems.

System of Expression	YEAST: *P. pastoris*	INSECT CELLS: Sf9	MAMMALIAN CELLS: FreeStyle™ 293-F Cells	
			Transient Production	Stable and Inducible Cell Line
**Plasmid**	**pPIC9K**	pFB1	pCI-neo	Lentivirus rLV.TRE3G
Expression mode	Integrative transformation in SMD1163	Infection by baculovirus	Transfection by electroporation	Lentivirus transduction
Induction of gene expression	Methanol	Viral infection		Doxycycline
**Construct**	**hERG(S1-coil)**			
Sequence	hERG(362-675) + coiled-coil	hERG(362-675) + coiled-coil	hERG(362-675) + coiled-coil	
N-ter tags	None	2-Strep + 3C	None	
Mutation	None	None	N589Q	
C-ter tags	10-His	TEV + 6-His	TEV + SBP + 6-His	
Number of AA	373 AA	407 AA	411 AA	
Monomeric protein	42,025 Da	45,701 Da	46,389 Da	
Tetrameric channel	168,099 Da	182,803 Da	185,555 Da	

**Table 2:** Binding values of [^3^H] dofetilide to hERG(S1-coil) expressed in 3 recombinant systems.

		Batch 1	Batch 2	Batch 3	Batch 4	Mean
**Yeast *Pichia pastoris***	**B_max_**	9.06 +/− 0.32	14.99 +/− 0.41			**12.03 +/− 0.37 pmol/mg**
	**K_D_**	10.77 +/− 1.08	9.98 +/− 0.68			**10.38 +/− 0.90 nM**
**Insect cells Sf9**	**B_max_**	1.57 +/− 0.03	0.50 +/− 0.04	0.90 +/− 0.14		**0.99 +/− 0.09 pmol/mg**
	**K_D_**	4.56 +/− 0.30	5.84 +/− 1.30	35.37 +/− 9.69		**15.29 +/− 6.92 nM**
**HEK293 transient**	**B_max_**	0.46 +/− 0.01	1.00 +/− 0.05			**0.73 +/− 0.04 pmol/mg**
	**K_D_**	4.54 +/− 2.24	7.53 +/− 1.00			**6.04 +/− 1.74 nM**
**HEK293 stable and inducible**	**B_max_**	3.71 +/− 0.27	6.36 +/− 0.14	15.13 +/− 0.53	6.07 +/− 0.38	**7.82 +/− 0.36 pmol/mg**
	**K_D_**	7.33 +/− 1.46	6.24 +/− 0.44	25.90 +/−2.20	11.03 +/− 2.02	**12.38 +/− 1.68 nM**

Saturation ligand binding experiments with [^3^H] dofetilide were carried out on membranes from yeast *Pichia pastoris*, insect cells Sf9 or mammalian cells HEK293 (transient transfection or stable and inducible cell line). Each Bmax and K_D_ values were calculated from triplicates of samples obtained from two to four batches of production in each recombinant system.

**Table 3:** Quantification of hERG functionality in *P. pastoris* and HEK membranes.

	*P. pastoris*	Stable and Inducible HEK	
	**Functional hERG in membranes**		
Method of quantification	Radioligand binding assay		
Channel concentration in crude membranes	9.06	6.36	pmol/mg of protein in membranes
Molecular weight of the tetrameric channel	168,099	185,555	g/mol
**Channel concentration in crude membranes**	**1.52**	**1.18**	µg/mg of protein in membranes
	**Total hERG in membranes**		
Method of quantification	Western blot		
Quantity of channel on Western blot	301.79	258.63	ng/lane
Quantity of total protein on Western blot	20	10	µg/lane
**Channel concentration in crude membranes**	**15.09**	**25.86**	µg/mg of protein in membranes
	**Percentage of functional channel in membranes**		
**Functional/Total**	**10%**	**5%**	

The total quantity of hERG in membranes of *P. pastoris* and stable HEK cells was calculated as illustrated in Figure 3. The functional quantity determined as shown in Figure 2. The percent of functional hERG in membranes was calculated as the ratio (*w*/*w*) of functional and total values.

## Supplementary Materials

The following are available online at https://www.mdpi.com/1422-0067/20/13/3181/s1.
