# Supplementary material for: Importance of the Choice of a Recombinant System to Produce Large Amounts of Functional Membrane Protein hERG
Source: Int J Mol Sci. 2019 Jun 28;20(13):3181. doi: 10.3390/ijms20133181 (PMC6651182; doi:10.3390/ijms20133181)
Supplement: Supplementary file 1 [file ijms-20-03181-s001.pdf]

Importance of the Choice of a Recombinant System to Produce Large Amounts of Functional Membrane Protein hERG

Lucie Vasseur[1], Thierry Cens[1], Renaud Wagner[2], Nathalie Saint[3], Valérie Kugler[2], Alain Chavanieu[1], Christine Ouvry[4], Clémence Dupré[4], Gilles Ferry[4], Jean A. Boutin[5]

[1] Institut des Biomolécules Max Mousseron, Université de Montpellier, Montpellier, France.

[2] Plateforme IMPReSs, CNRS UMR7242, Biotechnologie et Signalisation Cellulaire, Ecole Supérieure de Biotechnologie de Strasbourg, Illkirch, France

[3] PHYMEDEXP, Université de Montpellier, CNRS, INSERM, Montpellier, France,

[4] PEX Biotechnologie, Chimie & Biologie, Institut de Recherches SERVIER, Croissy-sur-Seine, France

[5] Institut de Recherches Internationales SERVIER, Suresnes, France

* Correspondence: jean.boutin@servier.com; Tel.: +33155724000

Figure S1: sequences of the wild type hERG

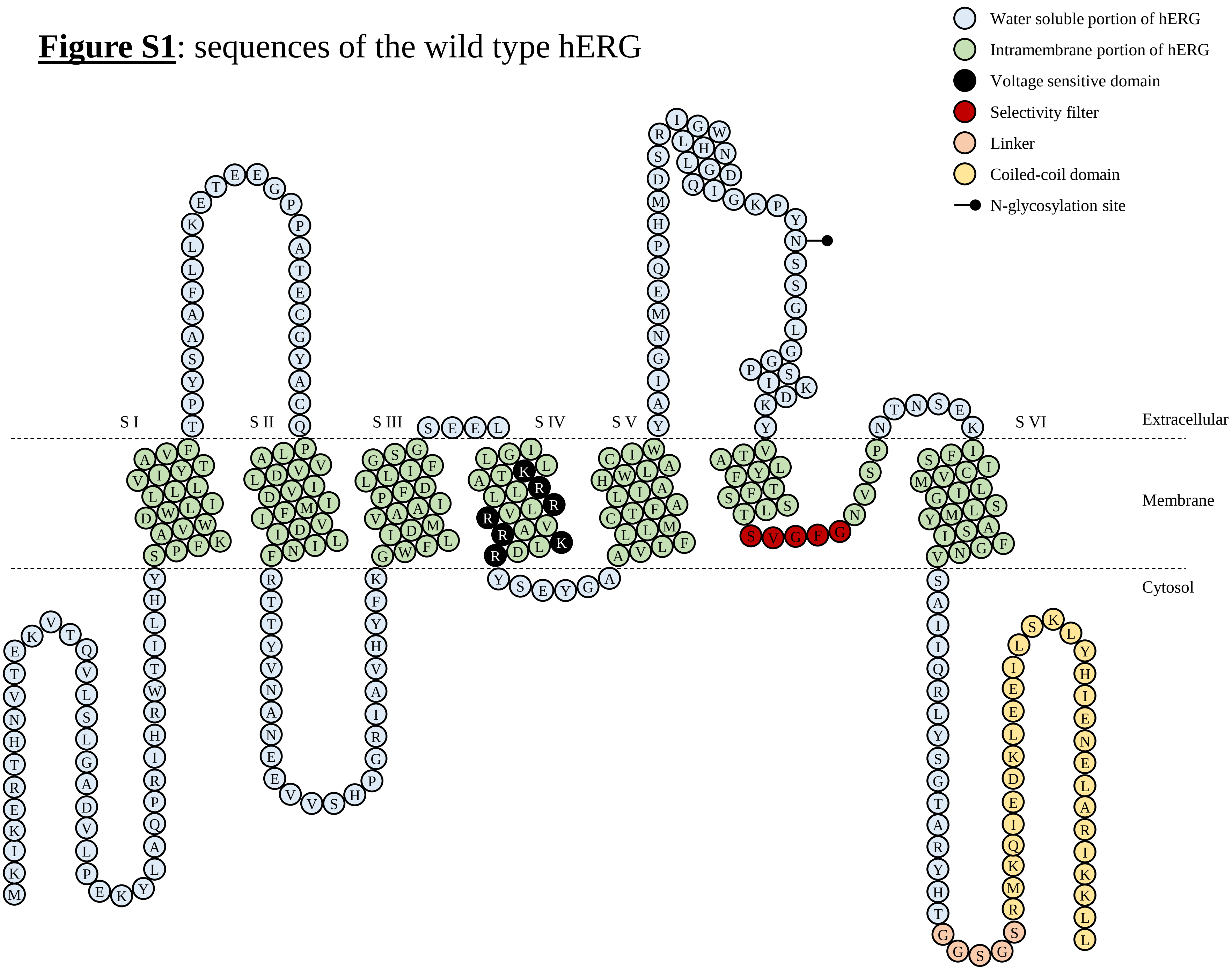

Figure S2: sequences of the hERG used in the present report

Amino acid sequence for expression in E. coli and Sf9

MWSHPQFEKGGGSGGGSGGSAWSHPQFEKGALEVLFQGPKIKERTHNVTEKVTQVLSLGADVLPEYKLQA
PRIHRWTILHYSPFKAVWDWLILLLVIYTAVFTPYSAAFLLKETEEGPPATECGYACQPLAVVDLIVDIMFIVDILI
NFRTTYVNANEEVVSHPGRIAVHYFKGWFLIDMVAAIPFDLLIFGSGSEELIGLLKTARLLRLVRVARKLDRYSE
YGAAVLFLLMCTFALIAHWLACIWYAIGNMEQPHMDSRIGWLHNLGDQIGKPYNSSGLGGPSIKDKYVTALYF
TFSSLTSVGFGNVSPNTNSEKIFSICVMLIGSLMYASIFGNVSAIIQRLYSGTARYHTGGSGSRMKQIEDKLEEI
LSKLYHIENELARIKKLLGERENLYFQGYDIHHHHHH*

Amino acid sequence for expression in HEK

MKIKERTHNVTEKVTQVLSLGADVLPEYKLQAPRIHRWTILHYSPFKAVWDWLILLLVIYTAVFTPYSAAFLLKE
TEEGPPATECGYACQPLAVVDLIVDIMFIVDILINFRTTYVNANEEVVSHPGRIAVHYFKGWFLIDMVAAIPFDLL
IFGSGSEELIGLLKTARLLRLVRVARKLDRYSEYGAAVLFLLMCTFALIAHWLACIWYAIGNMEQPHMDSRIGW
LHNLGDQIGKPYQSSGLGGPSIKDKYVTALYFTFSSLTSVGFGNVSPNTNSEKIFSICVMLIGSLMYASIFGNV
SAIIQRLYSGTARYHTGGSGSRMKQIEDKLEEILSKLYHIENELARIKKLLGERENLYFQGKLAAALEHHHHHH*

Amino acid sequence for expression in P. pastoris

MKIKERTHNVTEKVTQVLSLGADVLPEYKLQAPRIHRWTILHYSPFKAVWDWLILLLVIYTAVFTPYSAAFLLKE
TEEGPPATECGYACQPLAVVDLIVDIMFIVDILINFRTTYVNANEEVVSHPGRIAVHYFKGWFLIDMVAAIPFDLL
IFGSGSEELIGLLKTARLLRLVRVARKLDRYSEYGAAVLFLLMCTFALIAHWLACIWYAIGNMEQPHMDSRIGW
LHNLGDQIGKPYNSSGLGGPSIKDKYVTALYFTFSSLTSVGFGNVSPNTNSEKIFSICVMLIGSLMYASIFGNVS
AIIQRLYSGTARYHTGGSGSRMKQIEDKLEEILSKLYHIENELARIKKLLGERTSLERAPGGGSHHHHHHHHHH
*

(grey)	hERG 362-675
(pink)	Coiled coil
(yellow)	Strep tag
(green)	3C protease cleavage site
(blue)	TEV protease cleavage site
(red)	His tag
